# The effect of nurses’ organizational support on turnover intention: the chain-mediating role of psychological resilience and change fatigue

**DOI:** 10.3389/fpsyg.2025.1696053

**Published:** 2025-12-08

**Authors:** Zhenfan Liu, Xiaoting Yan, Zhitong Wang

**Affiliations:** 1 Department of Geriatrics, Deyang People’s Hospital, Deyang, China; 2 Department of Nursing, Sichuan Nursing Vocational College, Chengdu, China

**Keywords:** nurses, organizational support, turnover intention, psychological resilience, change fatigue, chain mediation effect

## Abstract

**Background:**

Nurse turnover intention remains high globally. Identifying targeted interventions to reduce turnover rates and stabilize the nursing workforce is a current priority. However, the mechanisms through which psychological resilience and change fatigue mediate the effect of organizational support on turnover intention are not fully understood.

**Objective:**

To investigate the current status of nurses’ turnover intention and to explore the mediating roles of psychological resilience and change fatigue between organizational support and turnover intention.

**Methods:**

In June 2025, 654 nurses were selected via convenience sampling. Surveys were conducted using the General Information Questionnaire, organizational support Scale, Connor–Davidson Resilience Scale (10-item), change fatigue Scale, and turnover intention Scale. A chain mediation model was constructed and validated.

**Results:**

The median scores (P25, P75) were as follows: turnover intention 8.00 (4.00, 10.00), organizational support 52.00 (49.00, 65.00), psychological resilience 24.00 (20.00, 30.00), and change fatigue 24.00 (20.00, 30.00). The direct effect of organizational support on turnover intention was significant (effect = −0.102). The separate mediating effects of psychological resilience and change fatigue, and their chain mediating effect, were −0.038, −0.030, and −0.010, accounting for 21.1, 16.7, and 5.6% of the total effect, respectively.

**Conclusion:**

Nurses’ turnover intention was at a moderately high level. The chain mediation effect of psychological resilience and change fatigue between organizational support and turnover intention was established. Management should focus on enhancing organizational support to improve psychological resilience, thereby mitigating change fatigue and ultimately reducing turnover intention, ensuring the delivery of high-quality patient care.

## 1 Introduction

Turnover intention refers to an individual’s contemplation of leaving their current job due to dissatisfaction and is a strong predictor of actual turnover (Feng et al., 2024). In 2021, the International Council of Nurses (ICN) projected a need for 10.6 million additional nurses by 2030 (Zhu et al., 2023). However, factors such as excessive workloads, negative work environments, inadequate compensation, and low social status contribute to high global nurse turnover rates (Zhang et al., 2022). A study in South Korea reported a nurse turnover rate of 15.4%, soaring to 45.5% for nurses with less than 1 year of experience (Korean Hospital Nurses Association, 2020). A cross-sectional study across five European countries found that 33% of nurses intended to leave their jobs (Ramoo et al., 2013). A meta-analysis in Ethiopia estimated the prevalence of nurse turnover intention at 53.35%, while a survey in China reported a rate of 69.4% (Elfios et al., 2024). As crucial components of the healthcare system, frequent nurse departures disrupt workforce stability, increase training costs, reduce organizational efficiency and care quality, and hinder the development of the nursing profession (Boateng et al., 2022). Addressing this challenge requires clarifying the factors influencing turnover intention and developing effective interventions.

Nurse turnover intention is influenced by various psychosocial factors, including organizational support (Dai and Ma, 2021). Organizational support reflects an individual’s belief that the organization values their contributions and cares about their well-being, encompassing both instrumental and emotional support (Battistelli et al., 2016). Grounded in social exchange theory, this perception fosters reciprocity, where nurses who feel supported exhibit higher job satisfaction and organizational commitment, thereby reducing turnover intention (Ho et al., 2021). Furthermore, the Conservation of Resources Theory (COR) posits that individuals strive to acquire and protect valuable resources, and organizational support, as a key job resource, can enhance nurses’ ability to cope with work-related stress (Hobfoll, 1989). When nurses perceive adequate organizational support, they are more capable and confident of maintaining their own resource reserves, thereby reducing the likelihood of resource depletion and, in turn, decreasing the subsequent turnover intention.

Psychological resilience, as a variable in positive psychology, primarily denotes nurses’ capacity for positive adaptation in the face of adversity (Troy et al., 2023). From the perspective of COR, psychological resilience can be regarded as a personal resource that enables nurses to effectively cope with resource loss or stressors in the workplace. Research has found that nurses with higher psychological resilience are more effective in coping with workplace challenges and demonstrate greater work enthusiasm, thereby reducing their turnover intention (Kuriyama et al., 2023). Meanwhile, psychological resilience acts as a facilitator of professional identity, effectively enhancing nurses’ professional identity, which in turn exerts a direct negative effect on turnover intention (Tao et al., 2025). Therefore, it is essential to highlight the direct negative relationship between nurses’ psychological resilience and their turnover intention. Additionally, relevant studies indicate a positive correlation between organizational support and psychological resilience—nurses who perceive organizational support exhibit higher levels of psychological resilience, experience further enhancement in work enthusiasm, and consequently, show reduced turnover intention (Shi, 2024). According to the Conservation of Resources (COR) theory, organizational support, as an external job resource, can enrich nurses’ personal resource pool and facilitate the formation and accumulation of psychological resilience—a core personal resource. In turn, psychological resilience helps individuals preserve existing resources and mitigate resource loss (e.g., burnout), thereby reducing turnover intention. Hypothesis 1 (H1): Organizational support negatively predicts turnover intention through psychological resilience (OS → PR → TI).

Change fatigue may represent another mediating variable. It describes the stress and exhaustion resulting from rapid, continuous workplace changes negatively impacting employees (Han and Guo, 2024). From the perspective of the Conservation of Resources (COR) theory, frequent organizational changes force nurses to invest additional energy and effort in adapting to new work requirements and the content of the changes. This directly depletes their personal resources, thereby leading to change fatigue. Nurses experience change fatigue more intensely than other healthcare professionals leading to job dissatisfaction and heightened turnover rates (Sarıgül and Uğurluoğlu, 2023; Fan et al., 2023). Organizational support can buffer this effect—according to COR, organizational support (a job resource) can compensate for the resource loss caused by organizational changes. When nurses feel supported by leadership, they maintain a more positive attitude toward change, reducing fatigue (Wang et al., 2023). Conversely, when nurses lack sufficient organizational support, their capacity to cope with the demands of ongoing changes becomes compromised. This leads to heightened levels of change fatigue, which in turn directly exacerbates job dissatisfaction and fosters the development of turnover intention. From the perspective of Social Exchange Theory (SET), the lack of organizational support breaks the reciprocal relationship between nurses and the organization. This makes nurses unwilling to invest excessive time and energy in adapting to organizational changes, which in turn increases their change fatigue and directly leads to their turnover intention. Hypothesis 2 (H2): Organizational support negatively predicts turnover intention through change fatigue (OS → CF → TI).

A randomized controlled trial (RCT) to examine whether psychological resilience training could alleviate change fatigue among nurses. The results demonstrated that enhanced psychological resilience significantly improved nurses’ levels of change fatigue. From the perspective of Conservation of Resources (COR) theory, psychological resilience—regarded as a personal resource—can help nurses reduce resource depletion caused by organizational changes, thereby alleviating organizational change fatigue. This conclusion is further corroborated by the study conducted by Brown et al. (2018): higher psychological resilience reduced change fatigue, thereby increasing nurses’ job satisfaction and subsequently decreasing their turnover intention. This reveals a sequential mediating mechanism: based on Social Exchange Theory (SET) and Conservation of Resources (COR) theory, organizational support effectively enhances nurses’ psychological resilience; in turn, grounded in COR theory, psychological resilience directly reduces change fatigue, ultimately lowering turnover intention. Hypothesis 3 (H3): Organizational support negatively predicts turnover intention through the chain mediation of psychological resilience and change fatigue (OS → PR → CF → TI).

In summary, nurses’ turnover intention is jointly influenced by dual organizational and individual factors, which has attracted widespread social attention. Although the academic community has conducted research on the individual mediating roles of psychological resilience and change fatigue, most existing studies only explore the direct relationships or single mediating effects between these variables. Research that integrates them into a chain mediation model based on multiple theoretical frameworks remains scarce—and this is precisely the key gap that this study aims to fill. Therefore, this study intends to analyze the current status of nurses’ turnover intention and its relevant influencing factors, while investigating the mediating roles of psychological resilience and change fatigue in the relationship between organizational support and turnover intention. This effort not only enriches the theoretical understanding of the formation mechanism of nurses’ turnover intention but also provides a more comprehensive explanation for how organizational support affects turnover intention. Furthermore, it expands the application of Conservation of Resources (COR) theory and Social Exchange Theory (SET) in the field of nursing management, ultimately laying a foundation for patients to receive high-quality nursing care.

## 2 Objects and methods

### 2.1 Participants

The study population comprised registered nurses working in tertiary and secondary hospitals in Sichuan Province, China. From this population, a sample of 654 nurses was recruited via convenience sampling in June 2025 from several of these hospitals. Inclusion criteria: (1) holding a valid nurse practicing license; (2) ≥ 1 year of clinical nursing experience; (3) informed consent and willingness to participate. Exclusion criteria: nurses on vacation or leave during the survey period. The sample size was calculated using the formula for quantitative studies (Ni et al., 2010): $n={\left(u_{a/2}\sigma /\delta \right)}^{2}$ , set *α* = 0.05, according to the standard deviation of the total score of the reference tendency to leave σ = 4.83, it is hoped that the allowable error δ is not more than 1, this study control δ value is set to 0.5, which results in *n* = 359, considering 20% of invalid questionnaires, the sample size of at least 431. The final analysis of this study was a sample of 654. The study was approved by the Institutional Ethics Committee (Approval No. 2023–04-070-K01).

### 2.2 Measures

All scales demonstrated good reliability. Confirmatory factor analysis indicated acceptable model fit for all scales (CFI > 0.90, TLI > 0.90, RMSEA < 0.08), supporting construct validity.

#### 2.2.1 General information questionnaire

Developed by the research team, it collected demographic data (gender, age, marital status, education) and work characteristics (hospital level, monthly income, professional title, department, years of experience, night shifts per week, weekly working hours, annual research output, daily professional learning time).

#### 2.2.2 Organizational support scale

This scale was developed in 2006 and revised in 2009, demonstrating high reliability and validity (Chen, 2006; Zuo, 2009). The Cronbach’s *α* coefficient of the original scale was 0.90. In their survey of nurses, Pu et al. (2025) reported a Cronbach’s α coefficient of 0.98. The scale comprises two dimensions: emotional support (Items 1–10) and instrumental support (Items 11–13), totaling 13 items. It employs a 5-point Likert rating scale, with responses ranging from “strongly disagree” (1 point) to “strongly agree” (5 points), yielding a possible total score range of 13 to 65 points. Higher scores indicate stronger perceived organizational support among nurses. In the present study, the Cronbach’s *α* coefficient of the scale reached 0.98.

#### 2.2.3 Connor–Davidson resilience scale (10-item)

Connor et al. (2003) developed a 25-item Resilience Scale, from which Campbell-Stills et al. extracted 10 items to form a shortened version. This shortened version was translated and revised into a Chinese edition, which demonstrated high reliability and validity (Wang et al., 2010). The Cronbach’s *α* coefficient of the scale was 0.91. In their survey of nurses, Li and Li (2015) reported an identical Cronbach’s *α* coefficient of 0.91. The scale employs a 5-point Likert scoring system, with responses ranging from “never” (1 point) to “always” (5 points). All items are positively scored, and the total resilience score is calculated by summing the 10 items, yielding a possible range of 10 to 50 points. Higher scores indicate greater psychological resilience among nurses. In the present study, the Cronbach’s *α* coefficient of the scale reached 0.98.

#### 2.2.4 Change fatigue scale

The scale was originally developed by Bernerth et al. (2011) and subsequently translated into Chinese and revised, demonstrating high reliability and validity. The Cronbach’s *α* coefficient of the original scale was 0.92. Comprising a single dimension with six items, this scale employs a 7-point Likert rating system, with responses ranging from “strongly disagree” (1 point) to “strongly agree” (7 points), yielding a total possible score range of 6 to 42 points. Higher scores indicate stronger levels of fatigue among nurses in response to organizational change. In the present study, the Cronbach’s *α* coefficient of the scale reached 0.94.

#### 2.2.5 Turnover intention scale

The scale was initially developed by Mobley et al. (1978) and subsequently translated into Chinese and revised, demonstrating high reliability and validity. The Cronbach’s α coefficient of the original scale was 0.93. This scale consists of a single dimension with four items, employing a 5-point Likert rating scale where responses range from “completely disagree” (1 point) to “completely agree” (5 points), resulting in a total possible score range of 4 to 20 points. Higher scores indicate stronger turnover intention among nurses. In the present study, the Cronbach’s α coefficient of the scale reached 0.94.

#### 2.2.6 Procedure

The research process was conducted as follows: First, the research team members compiled the questionnaire content, including the title, introduction, survey objectives, significance, methodology, and completion instructions. The questionnaire was then uploaded to the Wenjuanxing^1^ online survey platform to generate a dedicated survey link. Based on feedback from a pilot survey, the questionnaire was revised and finalized. Second, the project leader contacted department heads of relevant hospitals to explain the survey’s purpose, content, and significance. Upon obtaining their consent, these department heads were trained as survey administrators. They subsequently distributed the survey link and QR code to nursing staff via departmental WeChat workgroups during morning shift handovers or monthly training sessions. Finally, participants completed the questionnaire voluntarily after providing informed consent, with responses tailored to their individual circumstances. All items were mandatory, and each IP address was limited to one submission. A total of 686 questionnaires were collected. Responses with identical option sequences or completion times under 180 s or exceeding 1,200 s were deemed invalid. After excluding 32 invalid questionnaires, 654 valid responses remained, yielding an effective response rate of 95.3%.

### 2.3 Statistical analysis

Data were analyzed using SPSSAU. Descriptive statistics (medians, interquartile ranges [IQR] for non-normally distributed data) described sample characteristics and scale scores. Non-parametric tests (Mann–Whitney *U*, Kruskal–Wallis *H*) compared turnover intention scores across groups. Spearman’s correlation analyzed relationships between variables. Hayes’ PROCESS macro (v4.2, Model 6) tested the chain mediation model, using bootstrapping with 5,000 resamples to estimate 95% confidence intervals (CI). Statistical significance was set at *p* < 0.05.

## 3 Results

### 3.1 Sample characteristics

All 654 nurses completed the survey. Most were female (92.0%, *n* = 602), with a mean age of 33.86 ± 6.57 years. Most held a bachelor’s degree (80.9%, *n* = 529), were married (78.3%, *n* = 512), and worked in Grade III A hospitals (69.6%, *n* = 455). See Table 1 for detailed demographics and comparisons of turnover intention scores across groups. Significant differences (*p* < 0.05) in turnover intention were found across age groups, hospital levels, marital status, monthly income, professional title, position, weekly working hours, years of experience, departmental atmosphere, and annual research workload.

**Table 1:** Comparison of turnover intention scores by nurse characteristics.

Item	Variable	Number (%)	Score M(p25,p75)	H/Z	P
Hospital level	Tertiary A	455 (69.6%)	8.00(4.00,9.00)	18.499	<0.001
	Grade IIIB	70 (10.7%)	8.00(4.00,8.25)		
	Second Class A and below	129 (19.7%)	8.00(8.00,12.00)		
Age (years)	20 ~	187 (28.6%)	8.00(8.00,12.00)	10.447	0.015
	30 ~	346 (52.9%)	8.00(4.00,10.00)		
	40~	102 (15.6%)	8.00(4.00,9.00)		
	50~	19 (2.9%)	4.00(4.00,7.00)		
Marital status	Married	512 (78.3%)	8.00(8.00,12.00)	6.365	0.041
	Unmarried	123 (18.8%)	8.00(4.00,10.00)		
	Divorced	193 (2.9%)	8.00(6.75,12.00)		
Personal monthly income (yuan)	0 ~ <5,000	202(30.9%)	8.00(6.75,12.00)	33.259	<0.001
	5,000 ~ <8,000	268(41.0%)	8.00(4.00,10.00)		
	8,000 ~ <10,000	134(20.5%)	5.00(4.00,8.00)		
	>10,000	50(7.6%)	8.00(4.00,9.00)		
Professional title	Junior	315(48.2%)	8.00(4.00,11.00)	7.375	0.001
	Intermediate	296 (45.3%)	8.00 (4.00,10.00)		
	Advanced	43 (6.6%)	6.00(4.00,8.00)		
Position	None	583 (89.1%)	8.00(8.00,10.00)	7.432	0.024
	Associate Nurse Manager	20(3.1%)	4.00(4.00,8.75)		
	Nurse Manager and above	51(7.8%)	8.00(4.00,9.00)		
Mode of Employment	Establishment	112 (17.1%)	8.00(4.00,9.00)	−1.737	0.082
	Contracts	542 (82.9%)	8.00(4.00,10.00)		
Weekly working hours (hours)	0 ~ 40	195(29.8%)	8.00(4.00,9.00)	8.445	0.015
	40 > ~48	313(47.9%)	8.00(4.00,10.00)		
	>48	146(22.3%)	8.00(4.00,12.00)		
Years of working experience (years)	0 ~ <5	85(13.0%)	8.00(4.00,11.00)	9.717	0.021
	5 ~ <10	178(27.2%)	8.00(4.00,10.00)		
	10 ~ <20	311(47.6%)	8.00(4.00,10.00)		
	>20	80(12.2%)	6.00(4.00,8.00)		
Departmental atmosphere	Disharmony	14(2.1%)	12.00(8.00,16.00)	115.402	<0.001
	General	133(20.3%)	11.00(8.00,12.00)		
	Harmonious	507(77.5%)	8.00(4.00,8.00)		
Annual research output (items/year)	0 items	457 (69.9%)	8.00 (4.00,11.00)	8.694	0.013
	1 item	176 (26.9%)	8.00(4.00,8.00)		
	2 items and above	21(3.2%)	9.00(4.00,12.00)		

### 3.2 Descriptive statistics of key variables

Scores for the main variables are presented in Table 2. The median (IQR) scores were: turnover intention 8.00 (4.00, 10.00), organizational support 52.00 (49.00, 65.00), psychological resilience 24.00 (20.00, 30.00), and change fatigue 24.00 (20.00, 30.00).

**Table 2:** Scores of key variables (*n* = 654).

Variable	Items	Score range	Score, M (P25, P75)
Organizational support	13	13 ~ 65	52.00 (49.00, 65.00)
Emotional support	10	10 ~ 50	40.00 (37.00, 50.00)
Instrumental support	3	3 ~ 15	12.00 (12.00, 15.00)
Psychological resilience	10	10 ~ 50	24.00 (20.00, 30.00)
Change fatigue	6	6 ~ 42	24.00 (20.00, 30.00)
Turnover intention	4	4 ~ 20	8.00 (4.00, 10.00)

### 3.3 Comparison of turnover intention scores by nurse characteristics

The results of univariate analysis showed that the differences in the total scores of nurses’ turnover intention between different ages, hospital levels, marital statuses, individual monthly incomes, titles, positions, weekly working hours, years of working experience, unit atmosphere, and annual research workloads were statistically significant (all *p* < 0.05).

### 3.4 Correlation analysis

Spearman correlation analysis revealed the following significant relationships: Total scores and sub-dimension scores of nurses’ perceived organizational support were negatively correlated with change fatigue scores (*r* = −0.522 to −0.473, *p* < 0.05) and turnover intention scores (*r* = −0.595 to −0.573, all *p* < 0.05), while showing positive correlations with psychological resilience scores (*r* = 0.707 to 0.735, *p* < 0.05).

Psychological resilience scores were negatively correlated with both change fatigue scores (*r* = −0.467, *p* < 0.05) and turnover intention scores (*r* = −0.562, *p* < 0.05) change fatigue scores were positively correlated with turnover intention scores (*r* = 0.474, *p* < 0.05).

### 3.5 Chain mediation analysis

**Figure 1:**
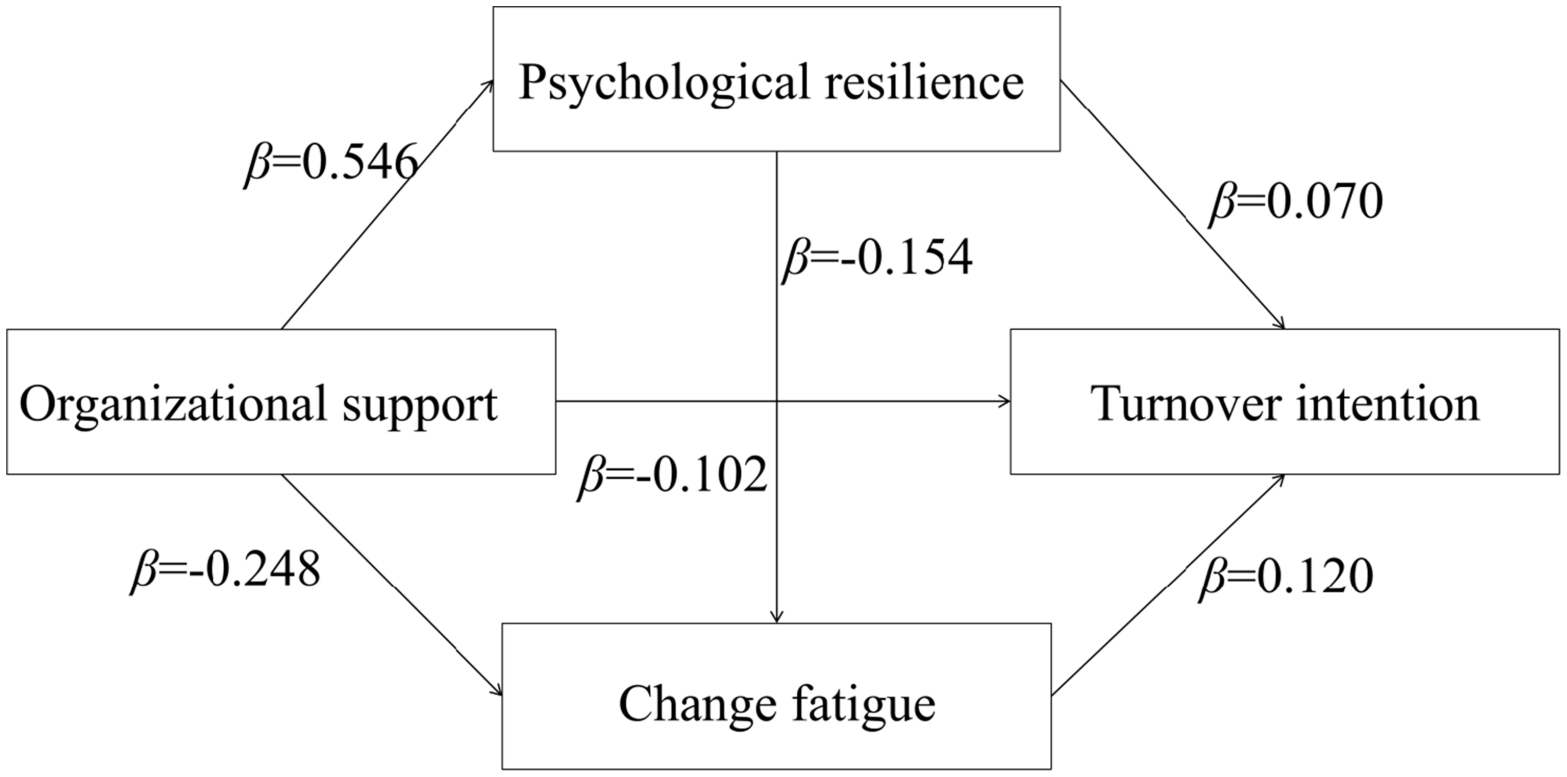
Chain mediation model of psychological resilience and change fatigue in organizational support and turnover intention among nurses.

The chain mediation effect was tested using Model 6 in the PROCESS macro. Control variables (age, gender, tenure, hospital level, etc.) were included in the model. Bootstrap method with 5,000 resamples was applied to calculate 95% confidence intervals (CIs). Mediation effects were considered significant when the intervals did not include zero. The results showed that: (1) organizational support positively predicted psychological resilience (*β* = 0.546, *p* < 0.001); (2) organizational support (*β* = −0.248, *p* < 0.001) and psychological resilience (*β* = −0.154, *p* < 0.001) negatively predicted change fatigue; (3) organizational support (*β* = −0.102, *p* < 0.001) and psychological resilience (*β* = −0.070, *p* < 0.001) negatively predicted turnover intention, while change fatigue (*β* = 0.120, *p* < 0.001) positively predicted turnover intention (see Table 3 and Figure 1 for details).

**Table 3:** Regression analysis for the chain mediation model.

Regression equation		Fit index			Significance of regression coefficients	
Outcome variable	Predictor variable	*R*	*R^2^*	*F*	*β*	*t*
Turnover intention	Organizational support	0.469	0.220	183.515	−0.180	−13.547**
Psychological resilience	Organizational support	0.652	0.425	482.432	0.546	21.964**
Change fatigue	Organizational support	0.377	0.142	53.982	−0.248	−5.647**
	Psychological resilience				−0.154	−2.929**
Turnover Intention	Organizational support	0.558	0.311	98.020	−0.102	−6.040**
	Psychological resilience				−0.070	−3.523**
	Change fatigue				0.120	8.161**

**P < 0.001.

The results of mediation analysis showed that the indirect effect of organizational support on turnover intention can be through 3 pathways: ① organizational support→ psychological resilience → turnover intention; ② organizational support→ change fatigue → turnover intention; ③ organizational support → psychological resilience → change fatigue → turnover intention. Among them, the total indirect effect accounts for 43.4%, including 21.1% of the mediating path ①, 16.7% of the mediating path ② and 5.6% of the mediating path ③ (see Table 4).

**Table 4:** Analysis of mediating effects.

Path	Standardized path effect	Effect size	95% CI	
			Lower	Upper
Organizational support – Psychological resilience – Turnover intention	0.546 × 0.070 = 0.038	21.1%	−0.060	−0.014
Organizational support – Change fatigue – Turnover intention	0.248 × 0.120 = 0.030	16.7%	−0.048	−0.015
Organizational support – Psychological resilience – Change fatigue – Turnover intention	0.546 × 0.154 × 0.120 = 0.010	5.6%	−0.019	−0.001
Indirect effects	0.038 + 0.030 + 0.010 = 0.078	43.4%	−0.100	−0.054
Direct effect	0.102	56.6%	−0.135	−0.069
Total effect	0.180	100.0%	−0.206	−0.154

## 4 Discussion

### 4.1 Levels of key variables

The results showed that nurses’ turnover intention scored 8.00 (4.00, 10.00) points, indicating a moderate-to-high level. This finding aligns with Zhao et al. (2024) but is higher than that reported for civil servants (Yang et al., 2019). The reasons may include: (1) 71.9% of nurses in this study had a monthly income below 8,000 RMB, with 30.9% earning less than 5,000 RMB, while 70.2% worked over 40 h per week. This reflects heavy workloads requiring excessive time and energy, yet personal income often failed to rise as expected, and career advancement was limited. Such conditions hindered the realization of self-professional value, leading to job burnout and subsequent turnover intention. (2) Data indicated that 70.2% of nurses worked night shifts at least once weekly. Long-term circadian rhythm disruption from night shifts not only triggers metabolic syndrome, reduced immunity, and other physiological issues but also exacerbates isolation by limiting interactions with family. This dual physical and psychological stress may create a vicious cycle, ultimately increasing burnout and turnover intention.

Organizational support for nurses scored 52.00 (49.00, 65.00) points, a moderate-to-high level, consistent with Lei et al. (2024). This may stem from: (1) sustained socioeconomic development enabling Chinese public hospitals to access greater social support and policy resources. These resources facilitate better working environments, intelligent equipment, and enhanced instrumental support for nurses. (2) Most nursing managers in general hospitals now undergo systematic training in professional knowledge and management skills, achieving basic modernization of nursing management. This has shifted management styles from one-way directives to bidirectional dialog, allowing nurses’ voices to be heard and fostering greater emotional support, thereby improving organizational support scores.

Psychological resilience among nurses scored 24.00 (20.00, 30.00) points, a moderate-to-low level requiring improvement. This phenomenon arises from the interplay of multiple stressors: (1) nurses’ constant exposure to patients’ pain, death, and other emotionally taxing scenarios inevitably impacts their mental health, leading to emotional exhaustion. (2) Despite organizational efforts to provide support and opportunities, heavy workloads leave nurses with limited time and energy for team activities or organizational interactions, resulting in low self-adjustment capacity and difficulty recovering from emotional exhaustion, thus lowering psychological resilience.

Change fatigue among nurses scored 24.00 (20.00, 30.00) points, a moderate level, higher than findings from Chinese scholar Yu et al. (2025) and Swedish scholar Fernemark et al. (2024). This discrepancy may relate to participant characteristics. Specifically: (1) 69.6% of nurses in this study were from tertiary Grade-A hospitals, which face frequent assessments and evaluations from higher authorities. This drives hospital management to implement policies requiring nurses to adapt to ongoing changes, with continuous organizational change directly inducing change fatigue. (2) Overloaded workloads and frequent night shifts further amplify fatigue when nurses face adjustments in work patterns (Lv et al., 2025).

### 4.2 Mediating role of psychological resilience

Psychological resilience partially mediated the relationship between organizational support and nurses’ turnover intention, with a mediation effect of 0.038, accounting for 21.1% of the total effect. This highlights a dual pathway: organizational support directly reduces turnover intention and indirectly influences it through psychological resilience (Poku et al., 2025). The findings of this study support the Conservation of Resources (COR) theory. Within the framework of this theory, organizational support—a type of job resource—helps foster psychological resilience, a personal resource. This enables nurses to better cope with various challenges, conserve emotional energy, and thereby reduce their turnover intention. Nurses with higher levels of psychological resilience can quickly adapt to work pressure and career challenges, effectively alleviate emotional exhaustion, and maintain a positive work state. Sufficient organizational support can further enhance psychological resilience by providing stress management training, emotional care, and a favorable work environment, thus significantly reducing turnover intention (Ike et al., 2024).

### 4.3 Mediating role of change fatigue

Change fatigue partially mediated the relationship between organizational support and nurses’ turnover intention, with a mediation effect of 0.030, accounting for 16.7% of the total effect. This finding supports Hypothesis 2 and is consistent with COR and SET. From the COR perspective, frequent organizational changes require nurses to invest a great deal of energy and resources to adapt, leading to resource depletion and change fatigue. Organizational support, as a job resource, can compensate for this resource loss, thereby reducing change fatigue and turnover intention (Hobfoll, 1989). From the SET perspective, when nurses perceive sufficient organizational support, they are more willing to cooperate with organizational changes and invest energy in adapting to them, reducing change fatigue and turnover intention. Conversely, the lack of organizational support breaks the reciprocal relationship, leading to increased change fatigue and turnover intention (Wang et al., 2023).

This result expands the understanding of the mechanism between organizational support and turnover intention from the perspective of change fatigue (Duan, 2024). As a key psychological load indicator in organizational change, change fatigue dynamically modulates nurses’ career development. When change-related pressure exceeds psychological tolerance thresholds, sustained organizational change may trigger emotional exhaustion and burnout, reducing work motivation and increasing turnover intention (Brown et al., 2018).

Moreover, effective organizational support not only optimizes change management processes by addressing nurses’ actual needs and minimizing non-essential adjustments—thereby reducing change fatigue at its source—but also enhances nurses’ adaptability through psychological support, skills training, and emotional care, alleviating existing fatigue. Additionally, a reciprocal relationship may exist between change fatigue and organizational support. A reduction in change fatigue may, in turn, strengthen nurses’ perceived organizational support, as those who adapt effectively to organizational changes are more likely to attribute their success to the support received. This fosters increased work engagement and ultimately diminishes turnover intention.

### 4.4 Chain mediation of psychological resilience and change fatigue

Psychological resilience and change fatigue jointly acted as chain mediators between organizational support and nurses’ turnover intention, with an effect value of 0.010, accounting for 5.6% of the total effect. This finding supports Hypothesis 3 and extends the application of COR and JD-R theory in the field of nursing management. According to COR, organizational support (a job resource) can promote the development of psychological resilience (a personal resource), and psychological resilience can help nurses reduce resource depletion caused by organizational changes, thereby alleviating change fatigue (Hobfoll, 1989). The JD-R theory posits that job resources can reduce the negative impact of job demands on employee outcomes by enhancing personal resources (Bakker and Demerouti, 2007). In this study, organizational support (a job resource) enhances psychological resilience (a personal resource), which in turn mitigates change fatigue (a job demand), ultimately reducing turnover intention. This chain mediation mechanism has rarely been explored in previous studies, which is a key contribution of this study. Most existing studies have focused on the single mediating role of psychological resilience or change fatigue, but few have integrated them into a chain mediation model. For example, Gao et al. (2025) found that resilience and change fatigue play a chain mediating role between intolerance of uncertainty and job burnout, but their study did not involve organizational support and turnover intention. This study fills this gap by verifying that organizational support can sequentially influence turnover intention through psychological resilience and change fatigue. This finding provides a more in-depth understanding of the psychological mechanisms underlying nurse turnover intention and suggests that improving psychological resilience can help nurses better cope with organizational changes, reduce change fatigue, and thus lower turnover intention.

## 5 Limitations

This study also has several limitations. First, the use of self-administered questionnaires may introduce common method bias in the reported results. Second, the convenience sampling method employed, with participants selected solely from hospitals in Sichuan Province, may limit the generalizability of the findings due to the region’s unique cultural and contextual characteristics. Third, the cross-sectional design can only examine associations between variables and does not permit causal inferences. Finally, the study did not incorporate objective indicators (e.g., actual turnover rates) to validate the measurements of turnover intention. Future research should: (1) Conduct cross-cultural, multi-center comparative studies to examine the applicability of this model across different healthcare systems; (2) Collect multi-source data (e.g., supervisor evaluations, objective staffing loss records) to enhance the robustness of the conclusions; and (3) Employ longitudinal or interventional experimental designs to verify the causal pathways among the variables. Based on these established relationships, targeted interventions should then be developed to reduce nurses’ turnover intention, encourage their more active engagement at work, and ultimately ensure the delivery of high-quality nursing care to patients.

## 6 Conclusion

The following conclusions were drawn from this study: Nurses’ turnover intention is at a moderately high level. Organizational support is directly and negatively correlated with turnover intention. The chain mediation effect of psychological resilience and change fatigue between organizational support and turnover intention is confirmed, supporting the integrated application of COR theory, SET, and the JD-R model. Adequate organizational support can enhance nurses’ psychological resilience, which helps them adapt to organizational changes and cope with change-induced fatigue, enabling them to maintain a positive work state and reducing turnover intention. These findings prompt managers to: (1) Enhance nurses’ sense of organizational support by providing a supportive work environment, reasonable remuneration, and engaging in regular communication to understand their difficulties and career development needs. (2) Prioritize improving nurses’ psychological resilience through mindfulness-based stress reduction courses, emotional management training, and fostering organizational care and team mutual support. (3) When implementing organizational changes, provide ample support and care for nurses. Cultivate psychological resilience to enhance their coping ability. Use appreciative inquiry approaches, consider the actual clinical situation and nurses’ needs, and reasonably arrange change plans to reduce change frequency and work pressure, thereby improving job satisfaction, reducing turnover intention, maintaining nursing team stability, and ultimately providing higher-quality patient care (Havens et al., 2006; Mistretta et al., 2018).

## Data Availability

The raw data supporting the conclusions of this article will be made available by the authors, without undue reservation.

## Footnotes

1
https://www.wjx.cn/
